# Predicting Early Atrial Fibrillation Recurrence Post-Electrical Cardioversion: A Critical Look at Bilateral Atrial Function

**DOI:** 10.3390/jcm14030749

**Published:** 2025-01-24

**Authors:** Fabio Anastasio, Guido Pastorini, Giacomo Pucci, Alessandro Gonella, Valentina Tardivo, Mauro Feola

**Affiliations:** 1 Cardiology Division, Regina Montis Regalis Hospital, ASLCN1, 12084 Mondovì, Italy; 2 Department of Medicine and Surgery, University of Perugia, 06123 Perugia, Italy; 3 School of Geriatry, University of Medicine, 10126 Turin, Italy

**Keywords:** electrical cardioversion, atrial fibrillation recurrence, antiarrhythmic drugs, mechanism of atrial fibrillation

## Abstract

**Background/Objectives**: The recurrence rate of atrial fibrillation (AF) after electrical cardioversion (ECV) appears to correlate with morpho-functional changes in both the left (LA) and right atria (RA). The present study focuses on identifying predictors for AF recurrence post-ECV. **Methods**: Sixty-one patients were included in the study following an elective ECV with a successful conversion to SR, and were subjected to cardiovascular assessment immediately after ECV. **Results**: At 6-month follow-up, 24 patients (39.3%) experienced AF recurrence. Patients without AF recurrence showed a lower right atrial valvular index (RAVi) (32 ± 8 vs. 40 ± 10 mL/m^2^, *p* = 0.03), a higher LA strain S-R (15.8 ± 7.7 vs. 9.0 ± 4.2%, *p* = 0.003), and more pronounced lateral a’ wave (5 ± 3 vs. 3 ± 1 m/s, *p* = 0.01), tricuspid a’ wave (7 ± 3 vs. 4 ± 2 m/s, *p* = 0.02), average a’ wave (6 ± 2 vs. 3 ± 1, *p* = 0.005), and augmentation index corrected for 75 beats per minute (Aix75) (26 ± 13 vs. 37 ± 12, *p* = 0.01). Based on these results, patients were assigned one point for each of the following criteria: RAVi > 36 mL/m^2^, average a’ wave > 4, LA strain S-R > 13%. The ROC curve analysis showed that a score of 3 had an AUC for AF recurrence of 0.81 (*p* < 0.001, CI 0.69–0.91), with a sensitivity of 96% and a specificity of 62%. **Conclusions**: LA strain, TDI Doppler, RAVi, and Aix75 measured immediately post-ECV were independent predictors of AF recurrence after ECV.

## 1. Introduction

Atrial fibrillation (AF) is the most common arrhythmia in the general population and is associated with significant cardiovascular morbidity. To improve the quality of life of people with AF, rhythm control is recommended, at least initially. However, the recurrence rate of AF within the first year after electrical cardioversion (ECV) ranges from 63 to 84%.

Numerous risk factors for AF recurrence have been identified, including older age, female sex, underlying cardiovascular diseases, smoking, pulmonary disease, and multiple AF episodes [1]. Morpho-functional changes in both left (LA) and right atria (RA) are also related to AF recurrence [2,3] and could be considered as intermediate markers of damage. Pathophysiologically, atrial inflammation and fibrosis have emerged as important factors associated with the development, progression, and recurrence of AF, due to their impact on electrophysiological and structural remodeling [4,5,6,7,8].

LA volume and LA strain were found to be inversely correlated with LA fibrosis [9,10]. Other factors associated with the failure of rhythm control strategies include atrial conduction wave [8,11,12], inter-atrial conduction time [13], p-wave dispersion [14], aortic stiffness [15], serum uric acid [16], epicardial fat thickness [17], laboratory tests [18], and use of statins [19].

This study aims to identify clinical, echocardiographic, electrocardiographic, hemodynamic, and laboratory parameters that affect the early recurrence of AF following the restoration of SR by electrical cardioversion.

## 2. Materials and Methods

### 2.1. Study Population

Between June 2022 and May 2024, 70 consecutive clinically stable patients underwent an elective ECV. Patients were included in the present study if they were characterized by age > 18 years, absence of history of cardiac surgery, absence of a pacemaker or implantable cardioverter-defibrillator, absence of moderate or severe valvular disease, good acoustic window, and successful ECV or absence of early recurrent AF (a recurrence before the discharge at 4 h).

ECVs were performed with a biphasic defibrillator between 100 and 200 Joules in patients under deep sedation. The decision to prescribe antiarrhythmic therapy with class Ic/III antiarrhythmic drugs after ECV was made after an accurate global evaluation of risk of AF relapse by an investigator blinded to the results of tissue Doppler assessment or other analyzed data. All patients received oral anticoagulation 3 weeks before and at least 4 weeks after ECV.

Medical history was defined by the Charlson Comorbidity Index (CCI). The previous use of antiarrhythmic drugs or other medications was recorded, as was other therapy and previous ECV. Lifestyle, physical activity, eating habits, and alcohol intake were recorded with a questionnaire. Participants were categorized as active versus inactive based on questions about participation in leisure-time physical activity over the past 30 days; those self-reporting 2 h per week of moderate-to-vigorous physical activity (MVPA) were classified as active. Blood samples for laboratory tests were taken for routine sampling before ECV in the Cardiology Intensive Care Unit. Twelve-lead electrocardiograms were obtained 2 h after ECV. All instrumental evaluations were performed 2 h after ECV and subsequently detailed.

The first recurrence of AF after the ECV was set as the primary end point. All patients were reassessed after 1 month and after 6 months. Information concerning AF relapses was obtained at 1 and 6 months after the ECV via review of electronic medical records of scheduled outpatient visits, 12-lead electrocardiograms, 24-hour ambulatory electrocardiographic monitoring, and hospital records.

### 2.2. Transthoracic Echocardiography

Comprehensive two-dimensional (2D) transthoracic echocardiography (TTE) and Doppler studies were performed by a single investigator (FA), using a General Electric VIVID E9 with an S5-1 1.5/3.6 MHz transducer (GE Healt Medical, Horten, Norway). All measurements were calculated over five cardiac cycles with a simultaneously obtained ECG. Two-dimensional measurements were assessed according to the American Society of Echocardiography and the European Association of Cardiovascular Imaging recommendations [20]. Two-dimensional biplane Simpson’s LVEF was used to assess left ventricular systolic function [21]. Myocardial deformation parameters—left ventricular global longitudinal strain (GLS) and peak atrial longitudinal strain (LA strain)—were obtained via off-line speckle tracking analysis using fully automated 2D speckle tracking software (Echopac 11, GE Healt Medical) for LV and LA longitudinal strain analysis. LA strain measurements were taken during the reservoir, conduit, and contractile phases of LA function denoted as left atrial strain reservoir function (LA strain S-R, LA strain S-CD and LA strain S-CT, respectively) [22]. Left and right atrial volume were calculated using the biplane disk summation technique using LA apical views and were indexed to the body surface area to obtain the LA (LAVi) and RA (RAVi) volume indexes. Mitral peak early (E) and late (A) diastolic filling velocities were recorded from the apical four-chamber view, with 3 mm of PW Doppler sample volume placed between the mitral leaflet tips. Pulsed-wave tissue Doppler imaging was used to obtain the myocardial velocities of the basal septal and lateral segments, as well as the right ventricular free wall, from the apical four-chamber view. An average of the three wave a’ was calculated. Atrial conduction time was measured from the p wave in the simultaneously obtained ECG to the Doppler wave [23,24]. The intra-observer variability for LVEF assessment was <5%. An LVEF value below 50% was considered indicative of left ventricular dysfunction.

### 2.3. Vascular Stiffness

After 10 min of rest with the subject in the supine position, blood pressure (BP) was measured in triplicate. Carotid-femoral pulse wave velocity (cf-PWV) was subsequently determined non-invasively (without changing body position) using the SphygmoCor system (SphygmoCor® Xcel; AtCor Medical, Sydney, Australia) through high-fidelity applanation tonometer to measure the pressure pulse waveforms at the carotid artery site, while a partially inflated cuff simultaneously obtained femoral pulse waves over the femoral artery at the leg midway between the hip and knee. The effective travel distance (ETD) was measured as 80% of the straight distance between carotid and femoral sites using a caliper, as recommended by an expert consensus on the measurement of aortic stiffness [25]. All PWV measurements were performed by a single trained operator (FA). The average of 10 different cardiac cycles on each of the sites was used for the analysis. Cf-PWV was automatically calculated according to the following formula: PWV (m/s) = path length (m)/transit time (s). Each PWV measure was conducted at least twice, with a 1-minute rest interval between each measurement. If the difference between PWV results was greater than 0.5 m/s, a third PWV measurement was performed. A brachial pulse waveform was also taken and analyzed by the SphygmoCor® device and used to derive the augmentation index, corresponding to the measure of the enhancement (augmentation) of central aortic pressure by a reflected pulse wave. AIx can then be defined as (P2-P1)/PP, where PP is the systolic–diastolic pressure difference and P2-P1 is the difference between the first systolic peak P1 (which is either the early systolic shoulder or the systolic pressure attributed to the forward pressure wave) and the second systolic peak P2 (augmentation pressure). AIx is standardized to a heart rate of 75 beats per minute (AIx75). Brachial waveform calibration to brachial SBP/DBP was performed before applying a generalized transfer function (GTF) to estimate a central BP waveform and obtain central SBP/DBP measurements [26].

### 2.4. Statistical Analysis

Statistical analysis was performed using SPSS Statistics 26 (IBM, Armonk, NY, USA). Categorical variables are presented as frequency with percentages and were compared using Fisher’s exact test. Continuous variables displaying normal distribution were expressed by mean ± standard deviation (SD), while values with asymmetric distribution were expressed as median [interquartile range (IQR)]. Categorical variables were expressed as absolute numbers and percentages. Continuous variables were analyzed with Student’s *t* test or Mann–Whitney U test and binomial variables with a chi squared test as appropriate. Bivariate correlations between two related variables were calculated using the Pearson’s or Spearman’s correlation coefficient (r). Univariate and multivariate logistic regression was performed to identify the potential predictors of AF recurrence. Variables with a *p* value < 0.05 in the univariate analysis were included in the multivariate analysis. Before multivariable analysis, to exclude any multicollinearity, preliminary analyses were performed by using the variance inflation factor (VIF), considering values between 1 and 10 as the absence of collinearity. Adjusted analysis was performed using age, sex, BMI, Charlson Comorbidity Index and the use of antiarrhythmic drugs as confounding factors. The results of the multivariable analysis are shown as the odds ratio (OR) with the corresponding 95% CI. A *p*-value < 0.05 was considered significant. The local ethics committee approved our study protocol, which complied with the Declaration of Helsinki.

## 3. Results

During the index period, 70 patients were evaluated. Nine patients (13%) were excluded for ECV failure or early recurrence (<4 h). Sixty-one out of 70 (87.1%) patients were converted to sinus rhythm and were included in the study (results).

The baseline characteristics of the 61 patients included in the study are summarized in Table 1. Patient age ranged from 41 to 80 years, with a mean of 66.4 ± 9.1 years. The median Charlson Comorbidity Index (CCI) was 3 [2,3,4]. Twenty-one patients had a previous positive medical history for past ECV attempt. Fourteen patients (22.9%) exhibited a reduction in left ventricular ejection fraction (LVEF) attributable to tachycardia-induced cardiomyopathy (TIC). Twenty-two patients were prescribed antiarrhythmic therapy (amiodarone or flecainide).

**Figure 1:**
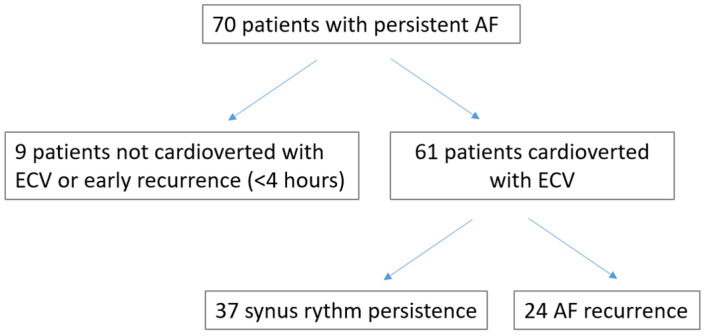
Flow chart of the study.

All patients underwent 1-month and 6-month re-assessment. Over a period of 6 months, 24 patients (39.3%) experienced an AF recurrence (Figure 1). Of these, 16 patients (26.2%) had AF recurrence over the first month, and eight (13.1%) between 1 and 6 months. There were no differences in anamnestic and anthropometric factors and laboratory tests between patients with or without AF recurrence at 6 months (Table 1). The two groups differed in terms of prevalence of physical activity (*p* = 0.02).

At the echocardiographic evaluation, patients with AF recurrence showed higher right atrial volume index (RAVi), higher LA strain S-R, higher lateral a′ wave, tricuspid a′ wave, and the average of the three a′ waves (all *p* < 0.05, Table 2).

Among vascular stiffness parameters, patients with AF recurrence, compared to patients without AF recurrence, had a higher augmentation index corrected for 75 beats per minute (Aix75) (*p* = 0.02, Table 3).

After correction for confounding factors, RAVi [OR 1.07 (95% CI 1.01–1.14), *p* = 0.02], LA strain S-R lateral [OR 0.70 (95% CI 0.57–0.86), *p* = 0.001], lateral a′ wave [OR 0.65 (95% CI 0.45–0.92), *p* = 0.02], tricuspid a′ wave [OR 0.72 (95% CI 0.55–0.94), *p* = 0.02], average of the three a′ waves [OR 0.62 (95% CI 0.44–0.88), *p* = 0.005], and Aix75 [OR 1.09 (95% CI 1.02–1.16), *p* = 0.01] were significant (Table 4).

**Figure 2:**
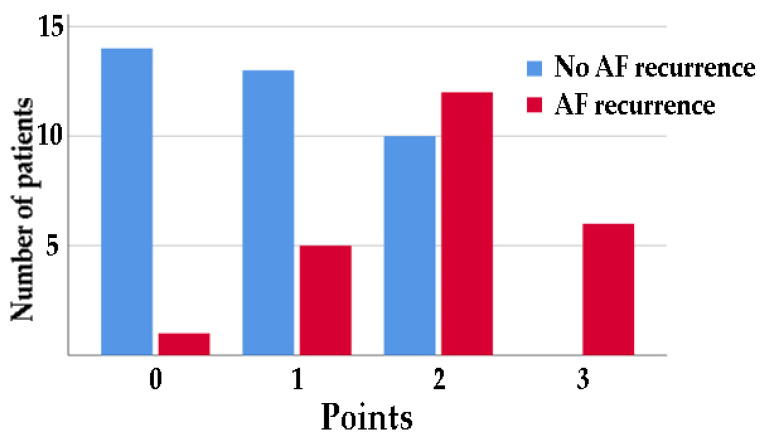
Number of patients with or without AF recurrence at 6 months divided by 1 point for each change above a cutoff of 36 mL/m^2^ for RAVi, below 4 cm/s for a′ wave average, and below 13% for LA strain S-R.

On the basis of these results, patients were divided into four groups based on a cutoff of 36 mL/m^2^ for RAVi, 5 cm/s for the tricuspid a′ wave, and 13% for LA strain S-R, with 1 point for each change above this value. Fifteen (24.6%) patients had 0 points, 18 (29.5%) patients had 1 point, 22 (36.1%) patients had 2 points, and six (9.8%) patients had 3 points (Figure 2).

**Figure 3:**
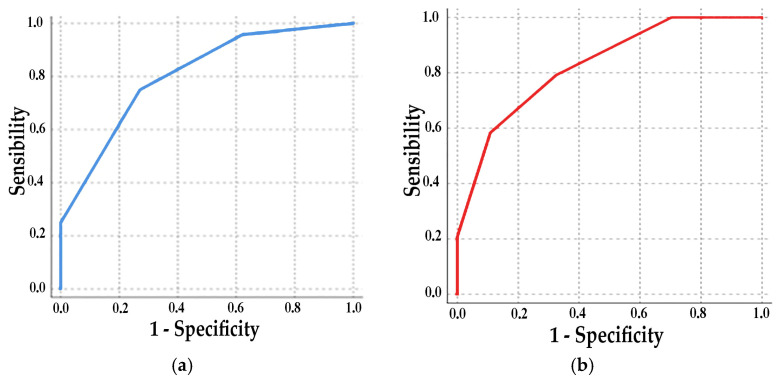
ROC curve: (**a**) 1 point for each change above a cutoff of 36 mL/m^2^ for RAVi, 4 cm/s for average a′ wave, and 13% for LA strain S-R; (**b**) 1 point added for Aix75 > 30.

Analysis of the ROC curve showed an area of 0.81 (*p* < 0.001, CI 0.69–0.91) with a sensitivity of 75% and a specificity of 73% at 2 points and a sensitivity of 96% and a specificity of 38% at 3 points (Figure 3a). Adding to this classification 1 point if Aix75 > 30, the ROC area increased to 0.83 (*p* < 0.001, CI 0.72–0.93) (Figure 3b). The comparative study between the parameters of the left and right atrium did not show a superiority of either.

## 4. Discussion

The present study investigated the prognostic value of immediate post-procedural cardiovascular assessment in predicting AF recurrence following ECV in patients with restored sinus rhythm. The main finding revealed that left atrial strain, biatrial function evaluated via TDI Doppler, and RAVi were independent predictors of AF recurrence post-ECV. Moreover, physical activity and the augmentation index exhibited a high degree of discrimination in identifying patients likely to experience AF recurrence after ECV.

### 4.1. AF Progression and Atrial Remodeling

Previous studies suggest that atrial electroanatomic remodeling, characterized by parietal fibrosis, alterations in ionic currents, and formation of reentry circuits, may contribute to the loss of atrial contractile function and their dilatation, thereby promoting the onset and maintenance of atrial fibrillation [4,5,6,7,8].

Several clinical risk factors for AF recurrence after ECV have been identified. Left atrial size is one of the most commonly utilized parameters for selecting patients for AF ECV [9]. However, the predictive accuracy of this parameter for the efficacy of ECV for AF is suboptimal [27]. In our study, LAVi did not correlate with AF recurrence, likely due to its greater association with LV diastolic dysfunction rather than direct atrial pathology. In contrast, two-dimensional speckle-tracking echocardiography of the LA has been shown to be a reliable method for assessing atrial function, demonstrating strong correlations with both histological atrial fibrosis and regional late gadolinium enhancement findings [10]. A more comprehensive estimation of the amount of atrial remodeling provided by LASr may be explained by the ability of speckle-tracking to detect subclinical injury of the LA myocardium independently of the LA volume [28]. In our study, LASr was significant associated with AF recurrence, although multivariate analysis was limited by collinearity with age, BMI, and CCI.

While several other factors, such as atrial conduction time, p-wave dispersion, aortic stiffness, and epicardial fat thickness, have been demonstrated to be valuable predictors of AF in patients undergoing either ECV or catheter ablation [11,12,13,14,15,16,17,18,19], these factors were not associated with recurrence of AF after elective ECV in our sample.

Concerning the right atrium, the role of the mechanics in the pathogenesis, progression, and treatment of AF has been less explored in the literature. RAVi was independently associated with incident AF and was a robust predictor of AF recurrence following either catheter ablation or ECV [29]. Recent reports also suggest that RA contractile function may play an additional role in AF recurrence, though RA strain is not as extensively studied as LA strain.

A direct expression of the contractile capacity of both atria can be obtained through TDI of the mitral and tricuspid annulus. The a’ wave, in particular, indicates the atrium’s contribution to ventricular filling, serving as a surrogate for atrial contractile function. Additionally, the a’ wave may be assess atrioventricular coupling. In our results, both tricuspid and mitral a’ wave were associated with AF recurrence, without a significant collinearity with confounding factors.

### 4.2. Clinical Implication

Atrial remodeling is a well-established risk factor for AF recurrence. However current AF management strategies do not take it into account when deciding how to treat AF. Nonetheless, atrial remodeling involves both the LA and RA similarly.

Our study demonstrates that the combined analysis of LA and RA size and function provides additional predictive value for AF recurrence in patients undergoing ECV.

Moreover, echocardiographic analysis of atrial function immediately post-ECV may be as effective as analyses performed days later to circumvent atrial stunning. Echocardiographic assessment of atrial function using tissue Doppler imaging (TDI) is easy, reliable, fast, widely available, and can be implemented in routine clinical practice when LA strain rate (LASr) analysis is not available.

AIx75 represents the pressure boost induced by the return of the reflected waves at the aorta. The superior prognostic role of AIx75 might stem from the effect of an earlier reflected wave on an impaired systolic function, causing left ventricle pressure overload. These data suggest that increased arterial stiffness and an early reflected wave could impose an additional load on left ventricular ejection, contributing to left ventricular/atrial remodeling. This potentially creates a vicious circle that favors the development and progression of heart failure [30] or influences LA pressure.

## 5. Conclusions

Results for bi-atrial function assessed via TDI Doppler, RAVi, and augmentation index acquired immediately post-CVE were independent predictors of AF recurrence after ECV. Specifically, in AF patients who achieved sinus rhythm after successful ECV, right atrial enlargement, a reduced a’ wave, and impaired LA strain were strong predictors of mid-term AF recurrence.

### Study Limitations

Some limitations of the present study must be acknowledged. Firstly, this is a single-center study conducted on a relatively small, selected group of hemodynamically stable patients with persistent AF. Secondly, the lack of a continuous monitoring device during follow-up may have led to undetected asymptomatic AF episodes. Although patients were encouraged to obtain an ECG upon experiencing palpitations to confirm AF, asymptomatic episodes may still have been missed. Further, larger multicenter studies with continuous monitoring are required to validate our findings and to validate the scoring model and assess its applicability. Thirdly, there was heterogeneity in the post-ECV drug treatments. In our study, patients with anti-arrhythmic drugs were not more likely to maintain SR, but we cannot exclude that their use may have slightly influenced our results. New studies are demonstrating the emerging role of agents such as SGLT2 inhibitors and GLP-1 receptor agonists in maintaining sinus rhythm and reducing AF recurrences [31]. This was not evaluated in our study. Furthermore, the presence of atrial stunning after ECV, which is usually most pronounced immediately after cardioversion and improves progressively over 4 to 6 weeks, may have influenced the results.

## Figures and Tables

**Table 1:** Clinical parameters of the study population.

	Total (n = 61)	No AF Recurrence (n = 37)	AF Recurrence (n = 24)	*p*
Sex (male)	51 (83.6%)	31 (83.8%)	20 (83.3%)	0.67
Age (years)	66.4 ± 9.1	66.2 ± 10.2	66.7 ± 7.5	0.97
BMI (kg/m^2^)	27.0 ± 5.4	27.1 ± 5.6	26.9 ± 5.1	0.88
Charlson Comorbidity Index	3 [2–4]	3 [2–5]	3 [2–3]	0.88
Hypertension	49 (80.3%)	30 (81.1%)	19 (79.2%)	0.61
Coronary artery disease	6 (9.8%)	3 (8.1%)	3 (12.5%)	0.54
Heart failure	11 (18.0%)	7 (18.9%)	4 (16.7%)	0.61
History of cardioversion	21 (34.4%)	13 (35.1%)	8 (33.3%)	0.94
Antiarrhythmic drug	22 (36.1%)	13 (35.1%)	9 (37.5%)	0.75
Class III anti-arrhythmic drug	18 (29.5%%)	10 (27.2%)	8 (33.3%)	0.34
Class IC anti-arrhythmic drug	4 (6.5%)	3 (8.1%)	1 (4.2%)	0.46
TIC	14 (22.9%)	10 (27.0%)	4 (16.7%)	0.48
Hemoglobin (g/dL)	14.3 ± 1.5	14.4 ± 1.4	14.1 ± 1.7	0.42
Creatinine (mg/dL)	1.0 ± 0.3	1.0 ± 0.2	1.0 ± 0.3	0.63
Potassium (mEq/L)	4.0 ± 0.4	4.1 ± 0.4	3.9 ± 0.3	0.13
Pro-BNP (pg/mL)	1537 [719–3063]	1118 [587–1976]	2521 [911–3550]	0.15
TSH (mU/L)	1.3 ± 0.6	1.6 ± 0.8	1.3 ± 0.5	0.24
Physical activity	13 (21.3%)	13 (35.1%)	0 (0%)	**0.02**
Alcohol consumption	44 (72.1%)	26 (70.2%)	18 (75.0%)	0.54
School	1 [1–2]	1 [1–2]	1 [1–2]	0.43
Income	4 [3–6]	4 [3–6]	4 [3–6]	0.62
AF recurrence at 1 month	16 (26.2%)			
AF recurrence at 6 months	24 (39.4%)			

Data are presented as mean ± SD or n (%) or median [IQR]. In bold are the statistically significant differences between patients with AF recurrences and no AF recurrences.

**Table 2:** Echocardiographic and electrocardiographic parameters of the study population.

	Total (n = 61)	No AF Recurrence (n = 37)	AF Recurrence (n = 24)	*p*
LVEDV (mL)	116 ± 29	119 ± 31	112 ± 27	0.54
SV (mL)	63 ± 13	65 ± 13	60 ± 12	0.25
LV mass index	100 ± 29	98 ± 26	105 ± 32	0.63
RWT (mm)	0.44 ± 0.08	0.44 ± 0.07	0.44 ± 0.09	0.86
GLS (%)	−15.6 ± 3.4	−16.3 ± 3.7	−14.6 ± 2.9	0.15
PSD (ms)	56.2 ± 22.2	54.7 ± 26.5	58.3 ± 15.5	0.48
LA volume (mL)	97 ± 34	101 ± 34	92 ± 34	0.46
LAVi (mL/m^2^)	50 ± 19	51 ±19	48 ± 19	0.64
LA strain S-R (%)	13.2 ± 7.2	15.8 ± 7.7	9.0 ± 4.2	**0.01**
LA strain S-CT (%)	−7.1 ± 5.1	−8.6 ± 5.3	−4.7 ± 3.4	0.06
LA strain S-CD (%)	−7.3 ± 5.8	−9.1 ± 6.5	−4.5 ± 3.4	0.07
RA volume (mL)	69 ± 19	64 ± 18	78 ± 18	0.06
RAVi (mL/m^2^)	35 ± 10	32 ± 8	40 ± 10	**0.02**
RV diameter (mm)	41 ± 4	41 ± 4	42 ± 4	0.84
TAPSE (mm)	20 ± 4	21 ± 3	20 ± 4	0.65
TV gradient (mmHg)	22 ± 6	21 ± 6	23 ± 5	0.24
Septal e/e′	9 ± 5	9 ± 6	9 ± 3	0.64
Lateral e/e′	10 ± 5	11 ± 6	10 ± 4	0.16
Tricuspid e/e′	8 ± 3	8 ± 3	9 ± 3	0.16
Septal a′ (cm/s)	4 ± 2	4 ± 2	3 ± 1	0.15
Lateral a′ (cm/s)	4 ± 3	5 ± 3	3 ± 1	**0.008**
Tricuspid a′ (cm/s)	6 ± 3	7 ± 3	4 ± 2	**0.009**
a’ average (cm/s)	5 ± 2	6 ± 2	3 ± 1	**0.004**
P wave (ms)	97 ± 34	97 ± 40	96 ± 28	0.68
Time p-a (ms)	156 ± 42	161 ± 60	149 ± 20	0.79
Time p-a′ septal (ms)	146 ± 56	147 ±71	144 ± 31	0.98
Time p-a′ lateral (ms)	155 ± 51	164 ± 65	142 ± 24	0.54
Time p-a′ tricuspid (ms)	131 ± 56	138 ± 67	120 ± 24	0.41
Epicardial Fat Thickness (mm)	7 ± 3	7 ± 3	7 ± 3	0.88
Presence of SVES	22 (36.1%)	12 (32.4%)	10 (41.7%)	0.32
P wave dispersion	42 (68.9%)	23 (62.2%)	19 (79.2%)	0.13

Data are presented as mean 6 SD or n (%). In bold are the statistically significant differences between patients with AF recurrences and no AF. Acronyms: LVEDV, left ventricular end diastolic volume; SV, stroke volume; LV, left ventricle; RWT, relative wall thickness; GLS, global longitudinal score; PSD, peak strain dispersion; LA, left atrium; LAVi, left atrial volume index; LA strain S-R, LA reservoir strain; LA strain S-CT; LA contraction strain; LA strain S-CD, LA conduit strain; RA, right atrium; RAVi, right atrial volume index; RV, right ventricle; TAPSE, tricuspid annular plane systolic excursion; TV, tricuspid valve; SVES, supraventricular extrasystole recurrences.

**Table 3:** Hemodynamic parameters of the study population.

	Total (n = 61)	No AF Recurrence (n = 37)	AF Recurrence (n = 24)	*p*
SBP (mmHg)	127 ± 17	124 ± 15	132 ± 20	0.24
DBP (mmHg)	77 ± 12	76 ± 10	78 ± 14	0.75
HR (beat/min)	63 ± 12	62 ± 12	65 ± 12	0.47
Central SBP (mmHg)	120 ± 19	118 ± 21	123 ± 18	0.55
Central DBP (mmHg)	77 ± 12	75 ± 11	78 ± 14	0.59
AIx75	30 ± 13	26 ± 13	37 ± 12	**0.02**
PWV (m/s)	11 ± 2	10 ± 2	12 ±2	0.13

Data are presented as mean ± SD. In bold are the statistically significant differences between patients with AF recurrences and no AF recurrences. Acronyms: SBP, systolic blood pressure; DBP, diastolic blood pressure; AIx75, aortic augmentation index corrected for heart rate 75; PWV, pulse wave velocity.

**Table 4:** Adjusted analysis of statistically significant parameters.

	OR (95% CI)	*p*	VIF
LA strain S-R	0.70 (0.56–0.87)	**0.003**	1.32
RAVi	1.07 (1.01–1.15)	**0.03**	1.08
Lateral a′	0.65 (0.45–0.92)	**0.01**	1.08
Tricuspid a′	0.72 (0.55–0.94)	**0.02**	1.07
A′ average	0.62 (0.44–0.88)	**0.005**	1.02
Aix75	1.09 (1.02–1.16)	**0.01**	1.06

In bold are the statistically significant differences between patients with AF recurrences and no AF recurrences.

## Data Availability

The original contributions presented in this study are included in the article. Further inquiries can be directed to the corresponding author.
